# Describing the musculature of mystacial pads in harbour seals (*Phoca vitulina*) using diceCT

**DOI:** 10.1111/joa.14158

**Published:** 2024-10-15

**Authors:** Alyx Elder, Elizabeth Evans, Charlotte Brassey, Andrew C. Kitchener, George Hantke, Robyn Grant

**Affiliations:** ^1^ Department of Natural Science Manchester Metropolitan University Manchester UK; ^2^ NXCT at the Henry Moseley X‐Ray Imaging Facility University of Manchester Manchester UK; ^3^ Department of Natural Sciences National Museums Scotland Edinburgh Scotland UK; ^4^ School of Geosciences University of Edinburgh Edinburgh UK

**Keywords:** diffusible iodine contrast‐enhanced computer tomography, extrinsic, harbour seal, intrinsic, mystacial pad, vibrissae

## Abstract

Pinnipeds have long, sensitive, moveable mystacial vibrissae. In other mammals, intrinsic muscles contribute to protracting the vibrissae. However, the mystacial muscles of pinnipeds have not yet been systematically described. Using traditional histological methods provides us with two‐dimensional muscle images, but having the ability to visualise these structures in three dimensions would allow for a more comprehensive understanding of pinniped vibrissal anatomy, especially given the challenges posed by their large and extremely curved mystacial pad. We predicted that harbour seals would have large, regular intrinsic muscles due to their well‐organised, moveable vibrissae. We adopted diffusible iodine contrast‐enhanced computer tomography (diceCT) to describe, for the first time, the three‐dimensional architecture of the mystacial vibrissal muscles found in harbour seals. Our observations show that their vibrissae are organised into grids within the mystacial pad. We identified both sling‐shaped and oblique intrinsic muscles that connect one vibrissae to the next in the same row. We also identified extrinsic muscles, including the m. nasolabialis, m. maxillolabialis, m. levator nasolabialis and m. orbicularis oris. Contrary to our prediction, the intrinsic muscles were not very large, although they were regularly distributed throughout the pad. Rather, the extrinsic muscles, particularly the m. nasolabialis and m. maxillolabialis were large, deep and well‐defined, running throughout the length of the mystacial pad. Therefore, we suggest that these extrinsic muscles, the m. nasolabialis and m. maxillolabialis, are responsible for driving vibrissal protraction underwater. These findings demonstrate the importance of three‐dimensional visualisation techniques in advancing our understanding of mystacial anatomy and function in pinnipeds.

## Graphical abstract

Harbour seals have movable mystacial vibrissae suggesting intrinsic muscles that aid protraction, yet their mystacial musculature is understudied. Using diceCT, we reveal the 3D structure of mystacial muscles for the first time. While the intrinsic muscles were regularly distributed, they were surprisingly small, contrary to expectations. Whereas the extrinsic muscles are large and deep, likely driving vibrissal protraction underwater. 3D imaging enhances our understanding of vibrissal anatomy, crucial for future studies on pinnipeds.

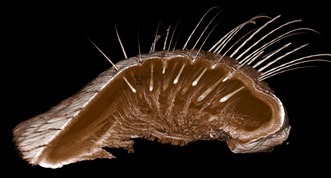

## 1 INTRODUCTION

Mystacial whiskers, or vibrissae, are specialised tactile hairs present on the faces of many mammal species (Ahl, 1986) and can vary between species in terms of size, shape, number and arrangement (Brecht et al., 1997; Dehnhardt, 2002; Muchlinski et al., 2020; Woolsey et al., 1975). Mystacial vibrissae are arranged in rows and columns (Woolsey et al., 1975) and are long, ordered and regular in nocturnal, arboreal and aquatic mammals (Grant et al., 2016, 2021; Muchlinski et al., 2020). Mystacial vibrissae guide behaviours, such as navigation, locomotion, exploration and hunting (Grant & Arkley, 2016; Grant et al., 2009, 2018). To do this, vibrissae are controlled and moved in complex patterns by facial muscles, bringing about changes in position, speed and spread (Grant et al., 2009; Mitchinson et al., 2007).

The muscles of mammalian facial vibrissae comprise of intrinsic and extrinsic muscles, which have been described across numerous terrestrial mammals, including mice, *Mus musculus* (Dörfl, 1982), hamsters, *Mesocricetus auratus*, (Wineski, 1985), guinea pigs, *Cavia porcellus* (Grant et al., 2016), opossums, *Monodelphis domestica*, (Grant et al., 2013b), brown rats, *Rattus norvegicus*, (Haidarliu et al., 2010), shrews, *Sorex unguiculatus* (Yohro, 1977) and nocturnal primates (Muchlinski et al., 2013). Intrinsic muscles are attached within the mystacial pad (Bosman et al., 2011; Dörfl, 1982; Haidarliu et al., 2010), whereas extrinsic muscles have their insertion points outside the mystacial pad (Dörfl, 1982; Haidarliu et al., 2010; Wineski, 1985). Both extrinsic and intrinsic muscles vary between species, although extrinsic muscles are thought to vary the most (Grant et al., 2013a; Yohro, 1977). The size and regularity of intrinsic muscles also differ between species (Grant et al., 2013a; Muchlinski et al., 2013). Nocturnal whisker specialists, such as mice and rats, have large, regular intrinsic muscles compared to more diurnal mammals, such as guinea pigs and short‐tailed opossums, which have smaller, less regular intrinsic muscles, that even cross between whisker rows (Grant et al., 2013b, 2016).

Pinnipeds are a group of marine carnivorans, comprising of phocids (true or earless seals), otariids (sea lions or eared seals) and odobenids (walruses). Pinnipeds are classed as whisker specialists due to the crucial role vibrissae play in their sensory ecology (Adachi et al., 2022; Dougill et al., 2020; Milne et al., 2020) and their vibrissae are highly sensitive (Dehnhardt et al., 1998; Hyvärinen, 1989; Hyvärinen & Katajisto, 1984; Marshall et al., 2006; McGovern et al., 2014). The most diverse vibrissae of any mammal are seen in pinnipeds (Dougill et al., 2020; Ginter et al., 2009), which vary between species, in terms of size, number, arrangement and shape, reflecting specialised adaptations for their aquatic lifestyles (Dougill et al., 2020; Ginter et al., 2009, 2012; Ling, 1977; Milne, et al., 2021a; Watkins & Wartzok, 1985). Distinctive vibrissal morphologies are notably different between the three families. Odobenids, otariids and three species of phocid—the bearded seal (*Erignathus barbatus*) and monk seals (*Monachus monachus* and *Neomonachus* spp.) possess smooth, vibrissae (Dehnhardt & Hanke, 2018); while all other phocid species have undulating vibrissae which are proposed to optimise underwater sensory perception by minimising signal‐to‐noise ratios during swimming (Hanke et al., 2010).

Phocid harbour seals (*Phoca vitulina*) have been extensively studied for their uniquely shaped undulating vibrissae and their role in sensory perception (Dehnhardt & Kaminski, 1995, 2001; Newby et al., 1970; Renouf, 1979a, 1979b; Wieskotten et al., 2010a, 2010b). Harbour seals have been shown to use their vibrissae to complete several discrimination tasks extremely quickly (<400 ms), detecting size differences down to 2 mm both on land and in water (Dehnhardt et al., 1995, 1998). In addition, harbour seals use their vibrissae for identifying different textures, being able to distinguish grooves with widths as small as 0.18 mm (Dehnhardt et al., 1998). Not only are harbour seal vibrissae well‐equipped for active touch sensing and object discrimination tasks, (Dykes, 1975), but using their vibrissae, harbour seals can detect and follow water movements of hydrodynamic trails (Dehnhardt et al., 2001; Wieskotten et al., 2010a, 2010b). They can extract critical directional information from just a single vortex ring, within a hydrodynamic trail, enabling them to determine swimming direction and effectively track prey (Krüger et al., 2018).

**FIGURE 1:**
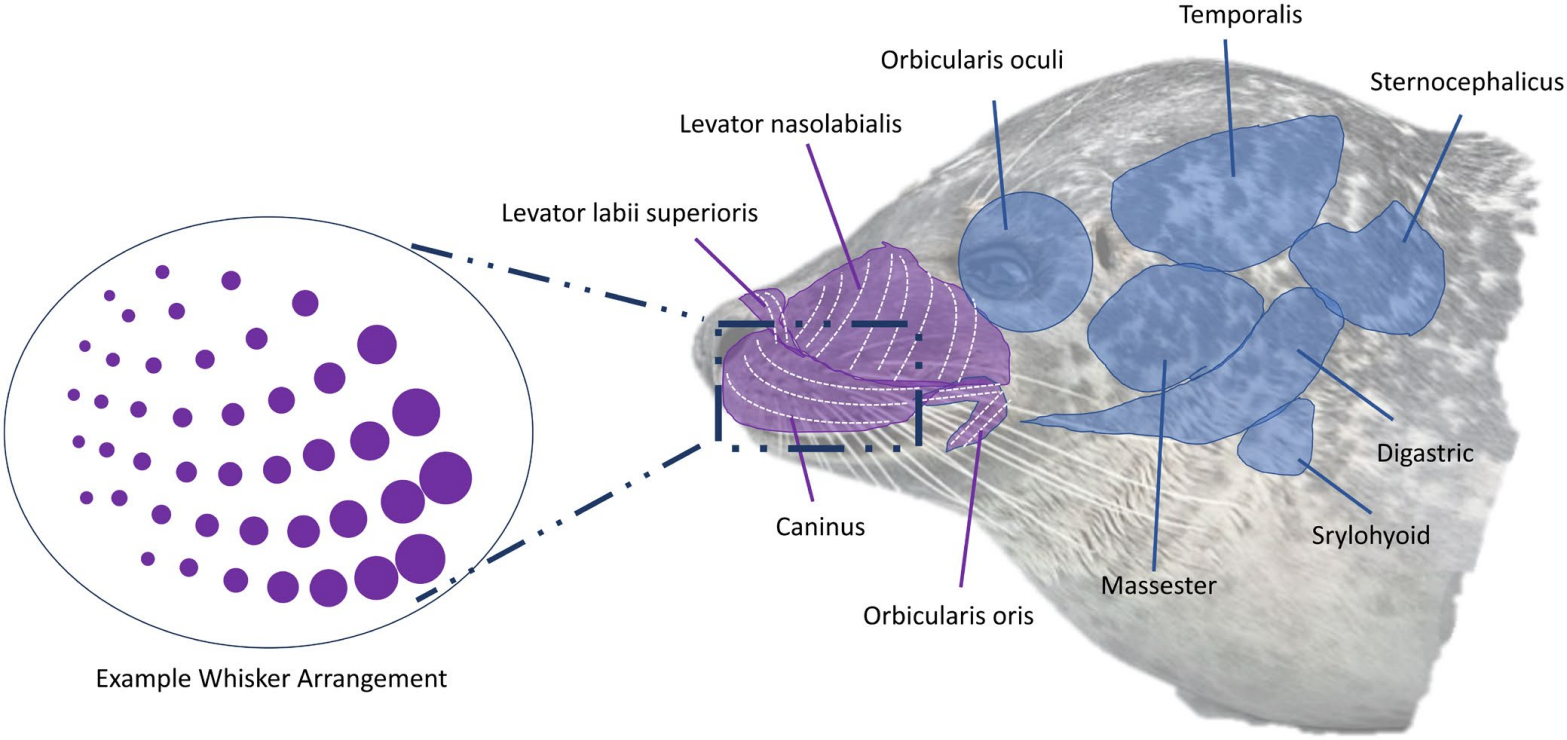
Harbour seal facial muscles, as described by Kienle et al. (2022): Showing muscles used for vibrissae movements within the mystacial pad (purple sections).

Diverse facial musculature in mammals significantly influences vibrissal movements during sensory exploration with vibrissae kinematics closely linked to facial musculature across various mammals (Grant et al., 2013, 2016; Haidarliu et al., 2010; Muchlinski et al., 2020). Unfortunately, when it comes to pinniped facial musculature, data are lacking in comparison to terrestrial mammals. Most of the facial musculature of pinnipeds appears to be associated with the base of the follicles and, in some pinniped species, erector pili muscles have been reported; possibly related to vibrissal mobility (Hyvärinen et al., 2009; Ling, 1966; Marshall et al., 2006). A recent study by Kienle et al. (2022) described the general facial muscles of several pinnipeds. Exploring the facial expression and mastication muscles in harbour seals, they identified several facial muscles that are linked to vibrissal movements in terrestrial mammals, including the caninus, orbicularis oris, levator nasolabialis and levator labii superioris, indicated by the purple sections in Figure 1, (Kienle et al., 2022). Dehnhardt and Hanke (2018) suggested that pinnipeds possess well‐developed intrinsic muscles that assist in protractions underwater but have not yet been described within the literature. Although pinnipeds do not continuously and rhythmically move their vibrissae, known as whisking, recent studies indicate pinnipeds, including harbour seals, can precisely control their vibrissae, suggesting specialised musculature in this species (Grant et al., 2023; Milne et al., 2020; Nakhwa et al., 2024).

While histology provides valuable information on vibrissal musculature, it is limited to two dimensions (2D) and damages the specimens (Sutton et al., 2013). Given the extreme curvature and large size of pinniped mystacial pads, visualising their vibrissal musculature in three dimensions (3D) would allow for a more comprehensive understanding of the pinniped vibrissal musculature anatomy required for vibrissal movements. Non‐destructive imaging techniques like CT scanning have improved our ability to examine 3D anatomical structures. Diffusible iodine contrast‐enhanced computer tomography (diceCT) enhances tissue contrast, offering high‐resolution 3D imaging and enabling precise digital segmentation for quantitative analysis, which is particularly useful for studying soft tissues like muscles (Gignac et al., 2014, 2016). Therefore, employing this technique would aid in describing the mystacial muscle architecture in pinnipeds, especially given the extreme curvature of their mystacial pads. The aim of this study is to describe, in 3D, the muscles within the mystacial pads of the harbour seal, enhancing our understanding of how pinnipeds control vibrissal movements. Other mammals with moveable and organised vibrissae have thicker and more regular intrinsic muscles (Grant et al., 2018; Muchlinski et al., 2020); therefore, we might expect that harbour seals will also have well‐developed and ordered intrinsic muscles within their mystacial pads, especially to overcome the drag associated with moving their vibrissae underwater.

## 2 METHODS

### 2.1 Mystacial pad preparation

Four harbour seal mystacial pads were loaned by National Museums Scotland. These specimens were recovered from dead, stranded animals on beaches in Scotland. Since the specimens are cadavers, to ensure accurate data acquisition, the mystacial pads were thoroughly examined to confirm all vibrissae were intact and that there was no visible damage to the pads. To preserve all the mystacial pads, chemical fixation was employed by immersion and storage in formalin solution (10%) at controlled temperatures (5–6°C). One specimen was removed from the fixing agent, rinsed in distilled water and stained using a buffered Lugol's iodine solution (2.5% concentration). The solution was prepared by dissolving 2.5 g of iodine and 5 g of potassium iodide (KI) in 100 mL of distilled water, which was then scaled up as necessary to fully immerse the specimen within an amber jar, to minimise the risk of staining agent degradation. The submersion period lasted for 28 days. Throughout the staining period, weekly monitoring of iodine levels facilitated precise control over the staining process and weekly preliminary tests were conducted to evaluate staining conditions throughout the specimen, to ascertain readiness for data collection. The preliminary tests on all mystacial pads consisted of fast, low‐resolution micro‐CT scans lasting <20 min, sufficient to determine if staining was constant throughout the mystacial pad, without shrinkage towards the pad exterior. All mystacial pads were allowed to equilibrate to room temperature overnight (approx. 12 h) before scanning, minimising potential movements or distortions during scanning under gravity, due to changes in temperature. Florist foam, masking tape and a clear, cylindrical low‐density plastic airtight container were used to house and support the mystacial pad while scanning. Once secured, mystacial pads were positioned in the scanner with the vibrissal area centred in the field of view, ensuring that the entire mystacial pad remained in the field of view during rotation. Before scans commenced, a preliminary movement test confirmed setup stability, preventing displacement during the rotation of the imaging plate. Once the movement tests were completed, scans were initiated and ran. All samples were uniformly prepared and scanned, so one specimen was chosen as a representative example. The selected example consisted of two separately dissected mystacial pads derived from a single specimen. This was selected by manually inspecting the quality of the CT scan data after testing multiple CT scan settings (see Evans & Elder, 2024 for full methods). Dividing the muzzle into two separate pads (left and right‐side cheeks) allowed for less digital pre‐processing before analysing the muscle structures, compared to processing the entire muzzle as a whole. The remaining three mystacial pads served as comparative references to validate the findings throughout and can be seen in the Supplementary Material. All procedures were approved by the local ethics committee at Manchester Metropolitan University (ID: 57712).

### 2.2 diceCT scanning

CT scanning was conducted at the University of Manchester, using the Nikon 225 kV *x*‐ray scanner called the ‘High Flux Bay’ at the Henry Moseley *x*‐ray Imaging Facility. The system included a 225 kV static tungsten reflection target source with a minimum focal spot size of 3 μm (at low wattage) and a PerkinElmer 4096 × 4096 pixels 16‐bit amorphous silicon flat‐panel detector, featuring a pixel pitch of 100 μm. A scanning protocol was employed, with various methods and techniques trialled and tested, as detailed in Evans and Elder (2024). The optimised scan parameters for the one stained specimen were set as follows: Voltage at 140 KV, current at 260 μA, wattage at 36.4 W, detector size at 2024 × 2024, gain 5, exposure at 1000 ms, number of projections at 3180, 4 frames per projection and voxel size at 38.56 μm. The scan duration averaged approximately 7 h. The 3D volumes were reconstructed from projection data at full resolution as 16‐bit TIFF stacks using Nikon's ‘CT Pro 3‐D software’ (Nikon Metrology, Tring UK). Beam‐hardening correction algorithms were applied during reconstruction to mitigate beam‐hardening artefacts, and no additional filters were employed. Data were exported utilising the full greyscale histogram range to facilitate comprehensive analysis.

### 2.3 Data processing and image data analysis

Volume files for datasets were cropped using ImageJ/FIJI (Rasband 1997–2018), to remove extraneous material. Further processing, analysis and visualisation were conducted using Avizo (Thermo Scientific™) versions 2019 and 2020.2. All data were analysed in 16‐bit greyscale to retain maximum image depth for segmentation based on grayscale value (Evans & Elder, 2024). Despite enhanced tissue contrast from iodine staining, significant overlap persisted in the mystacial pad. Staining procedures were restricted to 28 days to mitigate shrinkage risk, necessitating a combination of segmentation approaches. To reduce data noise, a non‐local means filter was applied to preserve edges and maintain boundaries (Gastal & Oliveiray, 2012). Manual and semi‐manual segmentation techniques were used to eliminate connected material (fur). The lasso (edge‐tracing) tool was applied slice‐wise for individual follicles, followed by interpolation, smoothing and comparisons with raw CT data. Major muscle groups, such as caninus muscle and levator nasolabialis (Figure 1), were approximated to represent general muscle boundaries within the mystacial pad. This involved manual segmentation of the linear trend on individual muscles every fifth slice in three orthogonal directions, followed by dilation, erosion, smoothing and a final round of erosion of the labels. Finally, intrinsic muscles were segmented using a histogram‐based manual segmentation approach. Throughout the process, data were analysed with recurring reference to the raw data and all mystacial pads for additional validation (Evans & Elder, 2024).

### 2.4 Second harmonic generation imaging

To confirm the presence of collagen within the harbour seal mystacial pads, two additional mystacial pads (M324/18 from Grant et al. (2023) and one unregistered) were loaned by National Museums Scotland, embedded in paraffin wax and sliced at 10 μm. Microscopy images were collected with a Leica SP8 TCS upright confocal microscope equipped with a HCX PL Fluotar 10× NA 0.3 objectives (Leica), 488 nm diode laser (Leica), MaiTai Deepsee multi‐photon laser (Spectra Physics) controlled by Leica LAS X software. Images were acquired in sequential mode. Second harmonic generation (SHG) imaging for collagen was excited with 880 nm line by multiphoton laser, and emissions were collected with HyD detector from spectrum range 435–445 nm; autofluorescence image was excited with the 488 nm line laser and emissions collected with PMT detector from spectrum range 500–600 nm. The image format was 1024 × 1024 pixels and for large‐area scanning, tile scan feature was applied. The images were saved as Lieca image file (.lif) and processed in Fiji (ImageJ) software.

### 2.5 Descriptive definitions

To enable interspecific comparisons, we adopted terminology from previous mystacial muscle studies in terrestrial mammals (Haidarliu et al., 2010) and assigned them to similar structures seen in the Harbour Seal, aligning them with terms used by Kienle et al. (2022) for pinniped facial muscles (Figure 1).

## 3 RESULTS

### 3.1 Vibrissae and follicle arrangements

**FIGURE 2:**
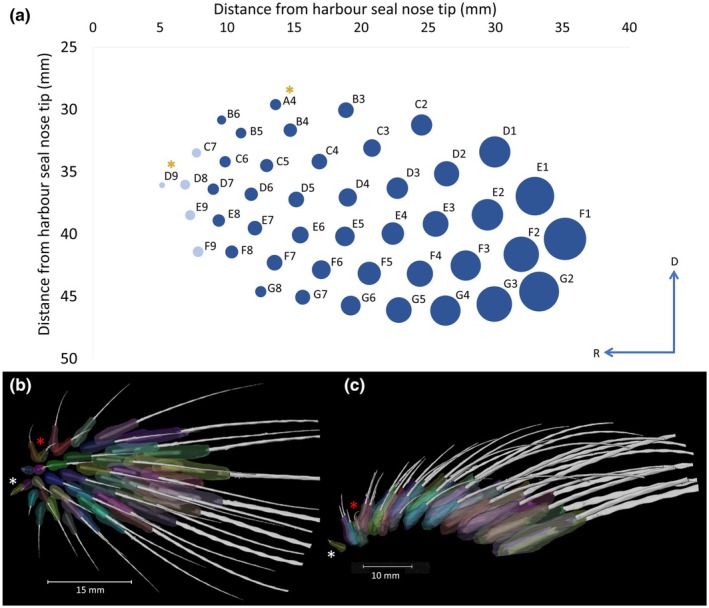
Quantitative analysis of vibrissae follicle distribution and arrangement on the harbour seal (*Phoca vitulina*) mystacial pad: (a) Rostral to caudal gradient of follicle volumes and their position within the mystacial pad. The size of each bubble corresponds to the follicle volume (mm^3^), starting with the smallest follicles in the rostral area and larger follicles towards the caudal area indicated by follicle volume <6 mm indicated by pale blue dots, yellow asterisk represents vibrissae lacking sling‐shaped intrinsic muscles, A4 and D9; (b) In the sagittal plane (from vibrissae tips to the interior of the mystacial pad cheek) (c) in the transverse plane (from the eyes down towards the chin), showing vibrissae and follicle arrangement, multicolours indicate separate follicles showing the organised grid‐like pattern in, with the red asterisk showing follicle B6 pointing rostrally out of the mystacial pad and the white asterisk showing the vibrissal hair shaft in D9 visible only in the follicle. Letters on the blue axis represent the direction of mystacial pad with ‘R’ rostral and ‘D’ dorsal.

Harbour seal mystacial pads contained a mean of 45 vibrissae, arranged in a grid‐like pattern (Figure 2 and Table S1, showing vibrissae counts for all mystacial pads). Typically, vibrissae were organised into seven rows on each side of the pad. For the stained specimen, row A contained a single vibrissa (A4), row B had four vibrissae (B3–B6) and row C consisted of six vibrissae (C2–C7). The three ventral rows (D–F) each contained nine vibrissae (1–9), while row G had seven vibrissae (G1–G7), totalling 45 vibrissae (Figure 2a and Table S1). The most caudal column of mystacial vibrissae, in some mammals, often displays follicles that straddle the vibrissal rows, referred to as a ‘Greek’ or ‘straddler’ arc, labelled with Greek letters, (Grant et al., 2013b, 2016; Haidarliu et al., 2010). However, harbour seals do not possess these ‘straddler’ vibrissae within the most caudal vibrissae column. Therefore, to align with the whisker arrangements in Belli et al. (2018) and Graff et al. (2024) we assigned the caudal‐most column as number one, with the remaining columns numbered consecutively through to nine.

The follicle volume exhibited a tenfold increase from the rostral columns, with follicle volumes as small as 1.97 mm^3^, to the caudal columns, with follicle volumes as big as 101.97 mm^3^ (Figure 2a). The rostral vibrissae C7, D8, D9, E9 and F9 had the smallest follicle volumes at <6 mm^3^. Indeed, rostral follicles were smaller compared to those in the centre and more caudal regions of the pad (Figure 2a–c). While there was no significant difference between follicle rows (dorso‐ventrally) (H (6) = 10.793, *p* = 0.09498), there was a significant difference between the follicle columns (rostro‐caudally) (H (9) = 42.304, *p* = <0.001). There was also a strong positive correlation between the follicle volume and the distance from the follicle to the nose tip (*S* = 482, *p* = <0.001), again suggesting that vibrissal follicles were smaller in the rostral area of the mystacial pad and increased gradually throughout, with the largest follicles towards the caudal area, shown in Figure 2. Follicles A4 and D9 lacked connections with the lateral extrinsic muscles and intrinsic muscles within the mystacial pad. The fact that these vibrissae sit outside of the mystacial pad could suggest that they are not strictly mystacial vibrissae and may be more similar to micro‐vibrissae (Figure 2a, yellow asterisk). Follicle B6 appears to point rostrally out of the mystacial pad (Figure 2c red asterisk). The vibrissal hair shaft in D9 appears absent in the superficial slices, suggesting possible shedding as it remains visible within the follicle when looking at the deeper data slices (Figure 2c, white asterisk).

### 3.2 Intrinsic muscles

**FIGURE 3:**
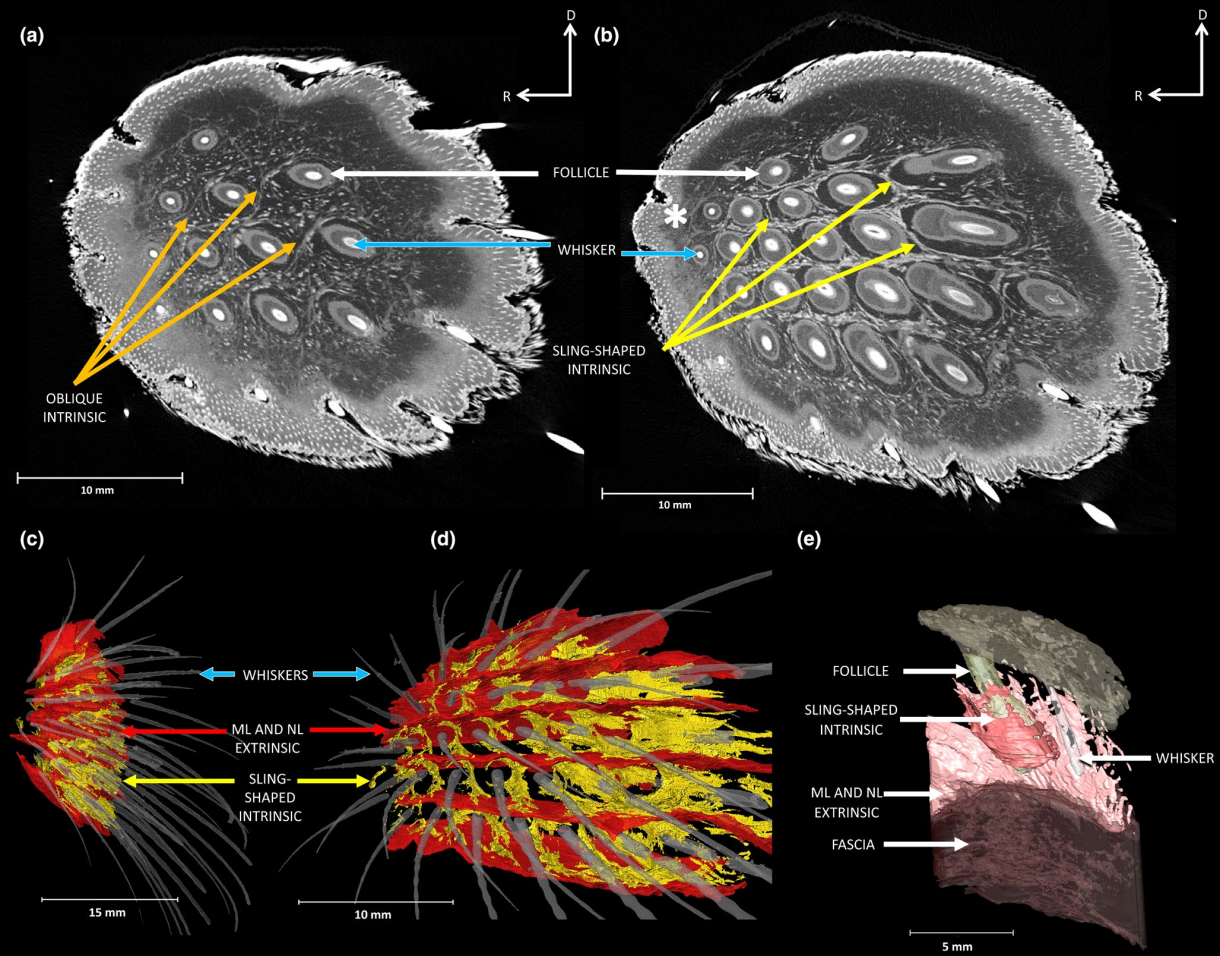
Harbour seal mystacial pad showing the intrinsic muscles: (a) Orthoslice in sagittal plane (from vibrissae tips to interior of mystacial pad cheek) showing oblique intrinsic muscles (orange arrows) located between follicles (white arrows) of the same row in the dorsal rows; (b) Orthoslice in sagittal plane of the sling‐shaped intrinsic muscles (yellow arrows) wrapped around the base of individual follicles, (white asterisk indicates less defined sling‐shaped muscles within the more rostral follicles); (c) From the frontal plane (nose through to interior of mouth), the intrinsic muscles surrounding each individual vibrissae (blue arrows) and extrinsic muscles (red arrows); (d) Segmentation in the sagittal plane of the vibrissae, sling‐shaped intrinsic muscles wrapped around the base of each individual vibrissal (yellow) and extrinsic muscles (red); (e) A close‐up of a segmented vibrissae section, displaying muscles and follicle in their raw form. Letters on the white axis represent the direction of mystacial pad with ‘R’ rostral and ‘D’ dorsal.

We observed both types of intrinsic muscles within the harbour seal mystacial pad—sling‐shaped and oblique intrinsic muscles. The sling‐shaped intrinsic muscles form a sling around the rostral area of each follicle and attach to the adjacent caudal follicle in the same row (Figure 3b and Figure S2 yellow arrows and Movie S1). These sling‐shaped intrinsic muscles can be clearly seen around all follicles in the pad, apart from A4 and D9 (Figure 2a, indicated by yellow asterisk), suggesting that these two follicles sit outside of the mystacial pad. By eye, the width of the sling‐shaped intrinsic muscles appears reduced and less defined, particularly around the rostral follicles in follicles C7, D8 and E9, (Figure 3b, indicated by white asterisk). All the sling‐shaped intrinsic muscles connect to individual vibrissae within the same row and can be seen clearly in all four mystacial pads (Figure S2, yellow arrows and Movie S1).

Oblique intrinsic muscles were observed in the dorsal rows B and C of the harbour seal mystacial pads (Figure 3a and Figure S2, orange arrows). In contrast to the sling‐shaped muscles, the oblique muscles connect the ventral capsular surface of the more rostral vibrissae follicles with the dorsal capsular surface of the more caudal vibrissae follicles in the same row. Figure 3a shows an oblique intrinsic muscle passing from the ventral part of B3 crossing to the dorsal part of B4 (Figure 3a and Figure S2b,d,f,h, orange arrows).

### 3.3 Extrinsic muscles

**FIGURE 4:**
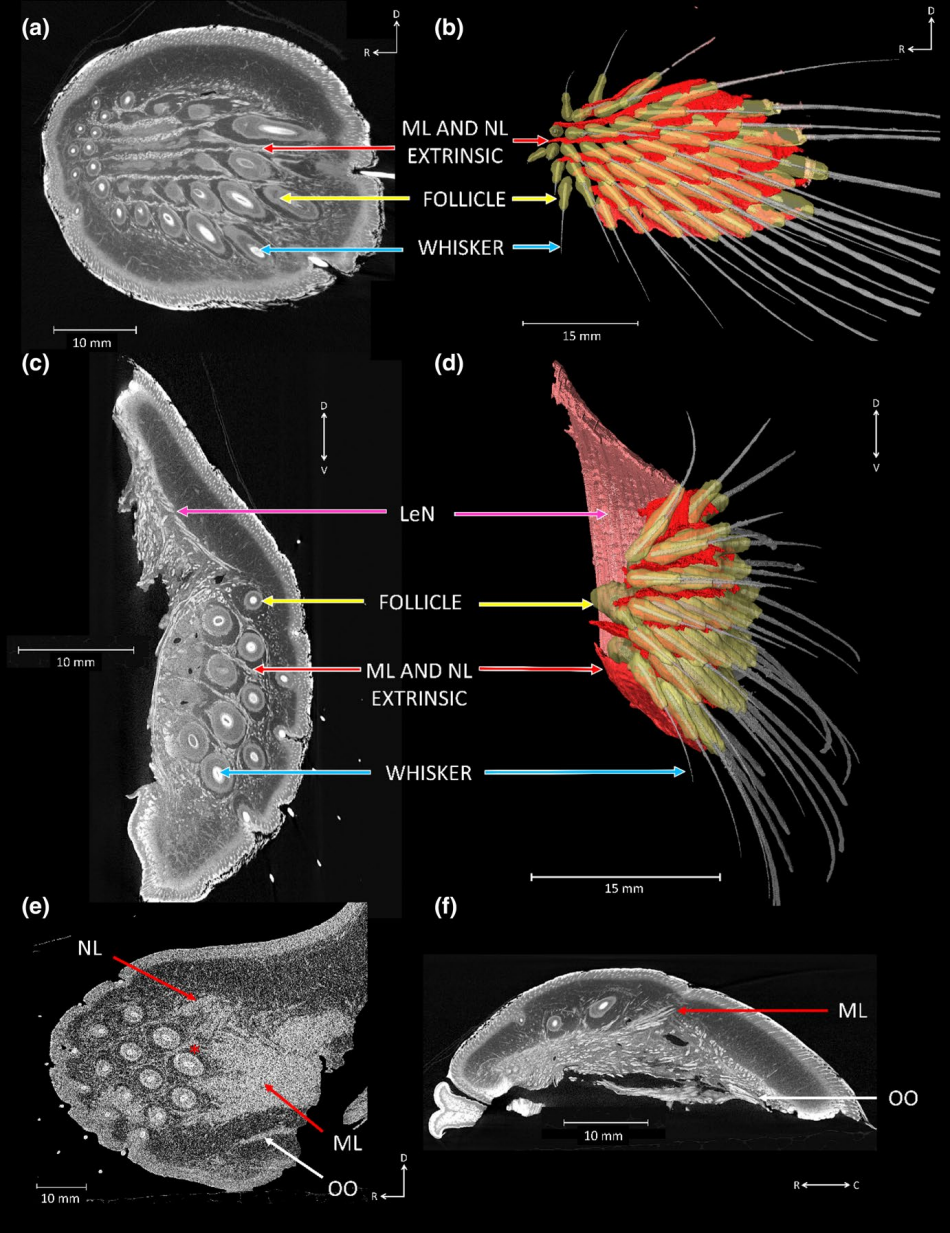
Harbour seal mystacial pad showcasing the extrinsic muscles: (a) Orthoslice in the sagittal plane (from vibrissal tips to interior of mystacial pad cheek) shows the extrinsic muscles m. nasolabialis (NL) and m. maxillolabialis (ML) (red arrows) located between the rows of vibrissae (blue arrows) and follicles (yellow arrows); (b) In the sagittal plane segmentation of the extrinsic muscles NL and ML (red) located between the rows of vibrissae white, follicles (yellow), surrounding individual vibrissae; (c) From the frontal plane (nose through to interior of mouth), we present the m. levator nasolabialis (LeN), (pink arrows), the extrinsic muscles NL and ML the vibrissae and follicles; (d) From the frontal plane segmentation of the extrinsic muscles NL and ML located between the rows of vibrissae, follicles surrounding individual vibrissae and vibrissae; (e) Image taken from a non‐iodine stained harbour seal mystacial pad, displayed in the sagittal plane showing the overlap of the ML and NL towards the caudal region of the mystacial pad (red asterisks) and the m. orbicularis oris (OO), (white arrows); (f) In the transverse plane (from eyes down towards chin), the extrinsic muscle ML and OO within the mystacial pad. Letters on the white axis represent the direction of mystacial pad with ‘R’ rostral, ‘C’ caudal, ‘D’ dorsal and ‘V’ ventral.

**FIGURE 5:**
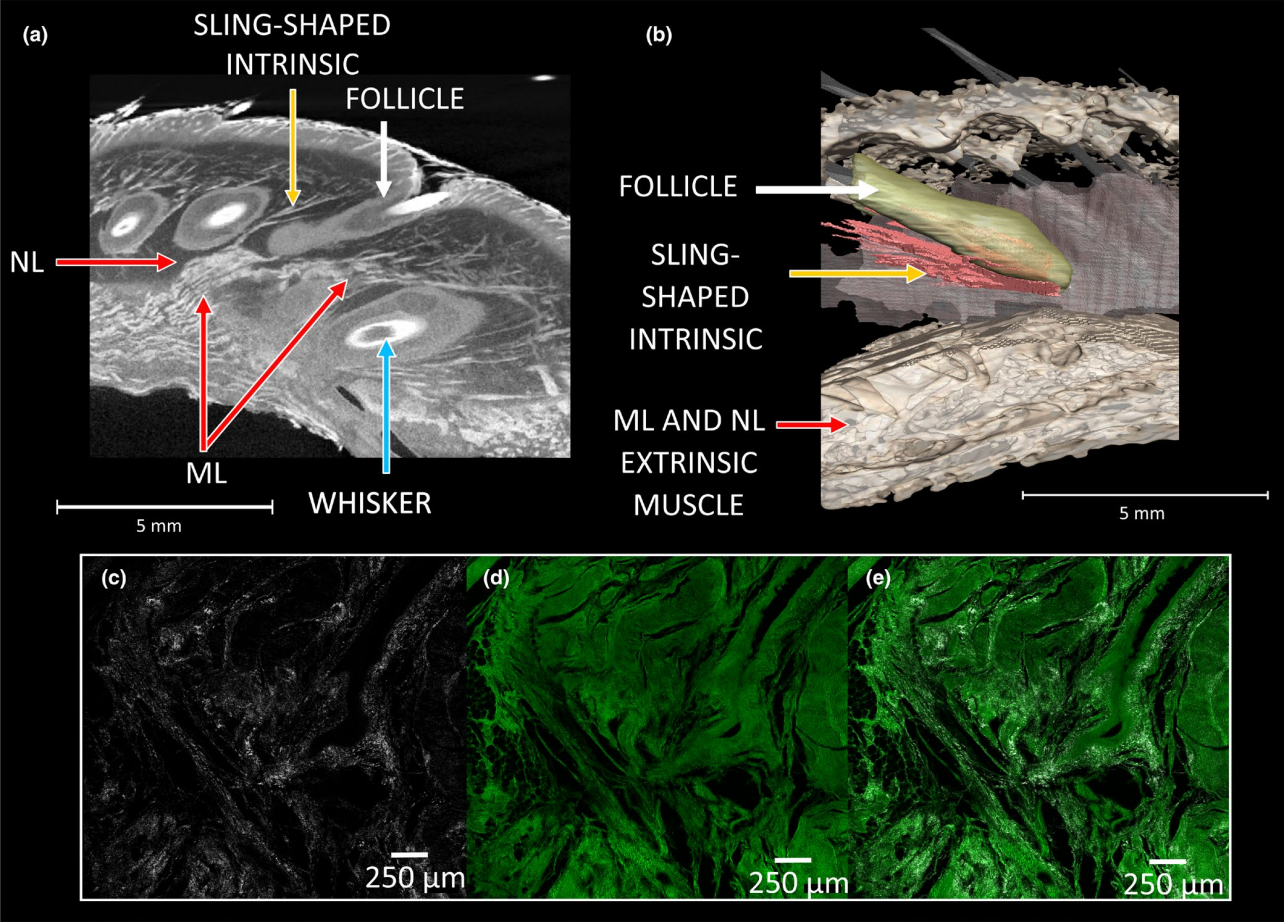
Extrinsic muscles in the harbour seal mystacial pad: (a) Orthoslice from the frontal plane (nose through to interior of mouth), showing the deep extrinsic muscles, NL and ML (red arrow) beneath the follicle allowing for vibrissal protraction; (b) Segmented raw vibrissal follicle displaying deep extrinsic muscles, NL and ML, with a partially segmented intrinsic; (c) Second harmonic generation (SHG) imaging of collagen excited with 880 nm line by multiphoton laser and emissions collected with HyD detector from spectrum range 435–445 nm; (d) Autofluorescence image excited with the 488 nm line laser and emissions collected with PMT detector from spectrum range 500–600 nm; (e) Overlay of c and d.

The extrinsic muscles, m. nasolabialis (NL) and m. maxillolabialis (ML) illustrated in Figure 4 (red arrows), can be clearly seen in all four harbour seal mystacial pads, (Figure 4 red arrows and Figures S1 and S2, red arrows). Within the pads, NL originates from the dorsal, caudal area of the pad and runs between the vibrissal rows before inserting deep into the mystacial pads fasciae. ML originates from the ventral caudal part of the mystacial pad and runs slightly deeper beneath NL (Figure 4e, red arrows), before running between each of the vibrissal rows. The NL and ML run dorsal to row A, between all rows, and are also found ventral to row, G (Figure 4a–e red arrows and Figures S1 and S2, red arrows and Movie S1). Their ends attach to the corium at the rostral border of the mystacial pad (Figure 4a,c,e, red arrows). Both these extrinsic muscles run via the corium and insert into the deeper layers of the mystacial pad into the fascia, between each vibrissal row (Figure 5a,b, red arrows). You can see from Figures 3e and 5a,b, that these extrinsic muscles run deeply beneath the vibrissal follicles within the pad.

Another extrinsic muscle identified in all four harbour seal mystacial pads was the m. levator nasolabialis (LeN). The LeN muscle runs dorsoventrally and was found especially in the rostral section of the pad (Figure 4c,d, pink arrows). The LeN inserts along the dorsal edge and is bordered ventrally by the caninus. Towards the ventral and caudal part of the LeN muscle, the m. orbicularis oris (OO) can be seen (Figure 4e,f, white arrows). The OO is a C‐shaped muscle, running along the ventral part of the pad, following the curve of the lip (Figure 4e,f). Bundles of the OO originate from within the fascia of the pad (Figure 4e,f). The presence of collagen was also confirmed throughout the mystacial pad. Collagen, which autofluorescences under SGH, was confirmed in the fascia, seen in Figure 5c,d.

## 4 DISCUSSION

We expected that harbour seals would possess large and organised intrinsic muscles, similar to those found in other whisker specialists, such as mice and rats; especially since harbour seal vibrissae need to overcome water drag to protract forward, leading us to expect even larger intrinsic muscles to facilitate this movement. However, although the intrinsic muscles in harbour seals are present and organised, their intrinsic muscles are not particularly large. Rather, the extrinsic muscles, particularly ML and NL, were extremely prominent, running between the vibrissal rows and follicles the whole rostro‐caudal length of the pad. These extrinsic muscles are so well‐defined; we suggest that they may play a crucial role in vibrissal movement and shaping the mystacial pad, particularly coordinating vibrissal movements to overcome the challenges of vibrissal protraction while underwater.

### 4.1 Vibrissae and follicle arrangements

The mystacial pad of the harbour seal has a distinctive grid‐like arrangement, characterised by organised rows and columns. This arrangement is commonly observed in nocturnal, arboreal and aquatic mammals, contrasting with the less organised and fewer vibrissae typically found in diurnal terrestrial mammals (Grant et al., 2018; Muchlinski et al., 2013, 2020). The most caudal column of mystacial vibrissae is often referred to as the ‘straddler’ vibrissae. It is primarily found in rodents and deviates from the regular grid pattern of mystacial vibrissae, appearing to ‘straddle’ the vibrissal rows, (Haidarliu et al., 2017). However, the harbour seal mystacial pad does not contain a distinct ‘straddler’ arc. Looking to see whether the intrinsic muscles straddle the caudal column is not hugely clear, but it is our opinion that the intrinsics of the neighbouring whiskers do not straddle the most caudal whiskers (Movie S2). Consistent with previous findings, our study confirms the presence of seven rows of vibrissae in the harbour seal mystacial pads (Dehnhardt & Kaminski, 1995; Jones & Marshall, 2019; Karpovich et al., 2022). Harbour seals possess a larger number of vibrissal rows compared to their terrestrial counterparts. For example, a rat has five vibrissal rows (Haidarliu et al., 2010). In our study, the number of vibrissae ranged from 42 to 47, with a mean of 45 vibrissae on each side of the mystacial pad (Table S1). This exceeds the range observed in terrestrial mammals, including the guinea pig, (23, Grant et al., 2016), opossum, (23, Grant et al., 2013a), hamster, (23, Haidarliu & Ahissar, 1997; Wineski, 1985), rat (30 Haidarliu et al., 2010), mouse (33, Dörfl, 1982) and shrews (40, Brecht et al., 2011). Although higher compared to terrestrial mammals, the number of vibrissae in harbour seals is similar to other pinniped species, including, 38 in the California sea lion (Dehnhardt 1994), 42 in the elephant seal (*Mirounga angustirostris*), (Smodlaka et al., 2017) and 44 in the grey seal (*Halichoerus grypus*), with considerably more vibrissae (121) seen in the bearded seal (*Erignathus barbatus*), (Marshall et al., 2006) and the walrus (350–700, Ling, 1977). The high number of vibrissae seen in pinnipeds may be associated with their underwater lifestyle, since they rely on their vibrissae for navigation, hunting and foraging in environments with limited visibility. This abundance of vibrissae enables them to detect hydrodynamic trails, flow patterns and disturbances caused by objects underwater, facilitating the detection of changes in water pressure, velocity and turbulence required for prey capture (Dehnhardt et al., 2001; Krüger et al., 2018; Wieskotten et al., 2010a, 2010b).

The size of vibrissal follicles within the harbour seal mystacial pad varied depending on their column position, with rostral follicles being smaller and caudal columns being larger (Figure 5). Some small rostral vibrissae did not have intrinsic muscles (Figure 2) and sat outside of the extrinsic muscle arrangements (Figure 3), suggesting they may not be mystacial vibrissae and may serve a different function in harbour seals, perhaps more like the microvibrissae of rodents. Rodents use their microvibrissae for detailed tactile sensing, dabbing them onto surfaces during object contact to gather more information (Brecht et al., 1997; Grant et al., 2011; Hartmann, 2001). Harbour seals may employ these micro‐vibrissae to detect small‐scale differences when exploring objects, with their placement at the rostral area of the mystacial pad enhancing the likelihood of effectively gathering tactile information, which has been noted before in Grant et al., (2013b).

### 4.2 Musculature of the mystacial pad

Harbour seals had both types of intrinsic muscles, the sling‐shaped and oblique. The presence of sling‐shaped intrinsic muscles is relatively unsurprising, as these are widespread among mammals with organised vibrissae and have been extensively documented across species, including in mice (Dörfl, 1982), hamsters (Wineski, 1985), guinea pigs (Haidarliu & Ahissar, 1997), opossums (Grant et al., 2013a), rats (Haidarliu et al., 2010), shrews (Yohro, 1977) and nocturnal primates (Muchlinski et al., 2013). Intrinsic muscles are primarily responsible for vibrissal protraction during exploratory behaviours in terrestrial mammals. Given that harbour seals have an organised layout of vibrissae, we anticipated that these seals would possess relatively large intrinsic muscles compared to the terrestrial whiskered specialists, like rats and mice. Given the increased resistance of water compared to air, we predicted that larger intrinsic muscles would be needed to protract the vibrissae. However, contrary to our expectations, the harbour seals' sling‐shaped intrinsic muscles had similar relative muscle widths (0.0002) to those seen in other mammals, including rats (0.0006), mouse (0.0017), guinea pig (0.0002) and opossum (0.0008). This suggests that the harbour seals did not have larger intrinsic muscles to enable protractions underwater, and we suggest that large m. nasolabialis and m. maxillolabialis extrinsic muscles are more likely to facilitate efficient protraction of the vibrissae underwater (see Table S2). Oblique muscles, which have been observed in opossums (Grant et al., 2013a), guinea pigs (Grant et al., 2016) and both rats and mice (Haidarliu et al., 2024), were also present within the more dorsal rows of the harbour seal mystacial pad, (row B and C). Oblique intrinsic muscles aid torsional rotation of the vibrissae (Grant et al., 2013a, 2016; Haidarliu et al., 2017). Therefore, the placement and attachment of the oblique intrinsic muscles imply their potential involvement in inducing torsional rotation of the dorsal vibrissae rows (B and C) during protraction. This would serve to orient the vibrissa, especially positioning the undulations at efficient angles of attack to improve hydrodynamic sensing. This coordinated interaction between the sling‐shaped and oblique intrinsic muscles ensures precise control of vibrissal movement, vital for effective tactile and hydrodynamic perception underwater (Dehnhardt, 2002; Milne et al., 2021b).

**FIGURE 6:**
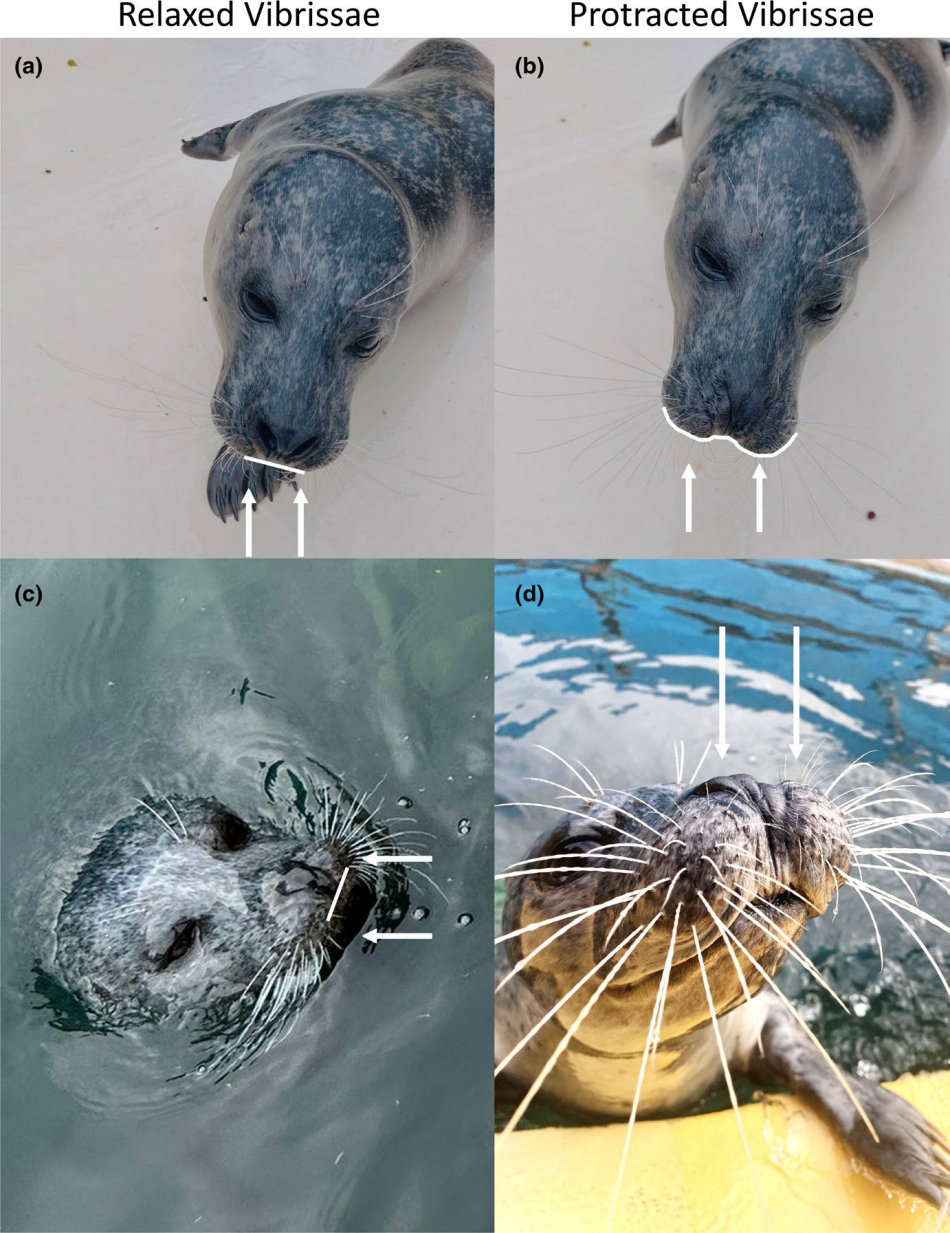
Visual representations of a harbour seal mystacial pad from relaxed to protraction of the vibrissae (a) Top‐down view showing relaxed vibrissae and a slightly relaxed mystacial pad, showing a flat pad across the nose (white arrows); (b) Top‐down view showing fully protracted vibrissae and a contracted mystacial pad indicated by the bulging cheek pads, especially around the nose (white arrows); (c) Top‐down view of relaxed vibrissae, showing a flattened pad while swimming and asymmetric whiskers (white arrows); (d) Frontal view of protracted vibrissae and increased vibrissal spread while exploring an object, with increase curvature and bulging of the cheeks and pad (white arrows).

Several extrinsic muscles were identified in harbour seals, including NL, ML, LeN and OO. In rats and mice, the NL and ML muscles function as vibrissal retractors; they are positioned superficially and pull the mystacial pad corium and distal ends of the follicles caudally, leading to vibrissal retraction and a reduction in vibrissal spread (Haidarliu et al., 2010). Conversely, we observe here, in the harbour seal, that the ML and NL extrinsic muscles are large and well‐defined. They appear to be deeper than those in rats and mice, as can clearly be seen in Figures 3e and 5a,b, where the fibres are deeper than the follicle base and extend up until around halfway up the follicle (Figure 3e). Therefore, we suggest that contraction of these muscles might cause protraction of the vibrissae. If this is the case, then we would expect several visible changes to occur when the muscles contract. Firstly, that the vibrissae would protract, the follicles would translate forwards and the pad would bulge around the snout. This can clearly be seen in Figure 6b,d, especially the bulging of the mystacial pad around the snout. This bulging of the pad observed here can be explained by hydrostatic deformation. In animal cells, changes in mechanical tension can lead to alterations in hydrostatic pressure, resulting in deformations that influence volume and shape (Chugh et al., 2022). This suggests that hydrostatic deformation seen in the pad could be a result of the coordinated contraction of both the intrinsic and extrinsic muscles. As these muscles contract, they create internal pressure within the pad, leading to changes its shape and volume. This forces the pad to bulge outwards around the snout, as seen in Figure 6b,d. Such controlled deformation could allow harbour seals to precisely adjust their vibrissae, enhancing tactile exploration, and optimise sensory input within underwater environments. Such an observation has not been documented for ML and NL muscles before, since they are superficially positioned in rats and mice, and usually cause a flattening of the pad (Haidarliu et al., 2024). The deep retracting muscles (Pars interna profunda (PIP), Pars maxillaris profunda (MP) and Pars maxillaris superficialis (MS)) have a similar effect in rodents but cause a retraction movement of the vibrissae, rather than a protraction movement, since they originate at the rostral area of the pad. Contraction of these deep retractors typically results in widening of the nostrils during retractions in rodents (Deschênes et al., 2015). We do not observe these muscles in our samples, and we also do observe changes in nostril opening coinciding with vibrissal movements (Figure 5), probably due to the deep ML and NL fibres originating from the caudal area of the pad, and not attaching to the nasal cartilage (unlike the deep retracting muscles in rodents). This is important, since harbour seals, like all pinnipeds, keep their nostrils naturally closed, preventing the incursion of water (Berta et al., 2006; Lilly, 1964). Overall, we think the presence and deep positioning of these well‐defined ML and NL extrinsic muscles could compensate for the less‐defined intrinsic muscles and may well be important in driving the protraction of the vibrissae underwater against drag and turbulence.

We also observed the extrinsic muscle OO, which plays several roles, including assisting with feeding, facilitating facial expressions and contributing to vocalisations (Kienle et al., 2022). Additionally, the OO plays a crucial role in controlling vertical vibrissal spread in pinnipeds, typically, pulling the vibrissae in the ventral rows downward (Kienle et al., 2022), which provides a larger tactile area for pinnipeds to explore (Figures 5e,f and 6d). However, Kienle et al. (2022) found that the OO muscle in harbour seals was not as well‐defined as those observed in other pinnipeds, such as bearded seal and Weddell seal (*Leptonychotes weddellii*). In these species, the OO muscle is enlarged, possibly due to specialised feeding adaptations, specifically, the ability to suction feed. However, several species, despite lacking adaptations for suction feeding, demonstrate proficiency in this strategy, including harbour seals (Kienle et al., 2018; Marshall et al., 2014). Harbour seals have been shown to change their feeding strategies when targeting different species of prey (Bowen et al., 2002). Therefore, having well‐developed facial musculature, multiple feeding behaviours and variability within feeding strategies is advantageous under different foraging scenarios and prey resources.

### 4.3 Limitations

The utilisation of 3D visualisation techniques provides valuable insights into mystacial anatomy. However, the application of diceCT scanning, while promising, is hindered by several limitations. This technique can give us insights into the musculature of pinnipeds and other vertebrates, yet it remains expensive and time‐consuming. Machine‐time expenses are substantial and larger samples require longer scan durations for high‐quality imaging results. Financial constraints limit the number of specimens that can undergo imaging, impacting data robustness. Additionally, contrast limitations within CT data hinder fully automated segmentation methods, leading to the necessity of manual segmentation processes and reduced repeatability. Low‐resolution scan configurations may compromise data quality, especially in capturing intricate structures. Challenges also persist in acquiring optimal carcasses for research purposes. Obtaining intact carcasses with preserved features (fully intact vibrissae) presents inherent difficulties and is particularly challenging due to numerous factors such as scavenging, post‐mortem procedures and transportation expenses. In addition, establishing appropriate staining protocols is crucial to preserve sample integrity, for example, preventing or reducing shrinkage, which is specifically important when looking at musculature. Despite these challenges, understanding musculature is essential for evolutionary and developmental insights. Thus, while complex and costly, addressing these limitations is crucial for advancing research in this field.

## 5 CONCLUSIONS

We describe details of the musculature throughout the mystacial pad in harbour seals using diceCT. Harbour seals possess ~45 vibrissae arranged in organised rows and columns, showing increasing vibrissal follicle volume from the rostral to the caudal region. Contrary to our original prediction, we found that harbour seals did not have large intrinsic muscles. Rather, the extrinsic muscles were notably large and well‐developed and may drive protraction movements in underwater environments helping vibrissae to overcome the drag and turbulence created by moving water. Considering there are variations in vibrissal morphology between pinnipeds, exploring vibrissal musculature in other species is essential for future research. Comparative analyses with other pinniped families, such as otariids and odobenidae, which have smooth conical vibrissae, in contrast to the undulating vibrissae of the harbour seal, may provide additional insights into the evolution of vibrissal functionality. By laying the groundwork for future investigations into pinniped musculature and tactile sensory capabilities, this research significantly enhances our understanding of the sensory adaptations of pinnipeds.

## AUTHOR CONTRIBUTIONS

**Dr. A Elder:** contributions to concept/design, acquisition of specimens and data, data analysis/interpretation, drafting of the manuscript, critical revision of the manuscript and approval of the article. **Dr. E Evans:** contributions to concept/design, data analysis/interpretation, drafting of the manuscript, critical revision of the manuscript and approval of the article. **Dr. C Brassey:** contributions to concept/design, acquisition of data, critical revision of the manuscript and approval of the article. **Dr. AC Kitchener:** acquisition of specimens and data, critical revision of the manuscript and approval of the article. **George Hantke:** acquisition of specimens and approval of the article. **Dr. R Grant:** contributions to concept/design, data analysis/interpretation, drafting of the manuscript, critical revision of the manuscript and approval of the article.

## CONFLICT OF INTEREST STATEMENT

Authors declare that they have no conflict of interest.

## Supporting information


Data S1:

Movie S1:

Movie S2:

## Data Availability

The data that support the findings of this study are available from the corresponding author upon reasonable request. Users must specify use and agree to commercial use restrictions. Requests will be reviewed by the PI.
