# Supplementary material for: Describing the musculature of mystacial pads in harbour seals (Phoca vitulina) using diceCT
Source: J Anat. 2024 Oct 15;246(5):696–708. doi: 10.1111/joa.14158 (PMC11996717; doi:10.1111/joa.14158)
Supplement: Supplementary file 1 — Data S1: [file JOA-246-696-s003.zip › Supplementary Material - Elder et al.pdf]

Supplementary Material

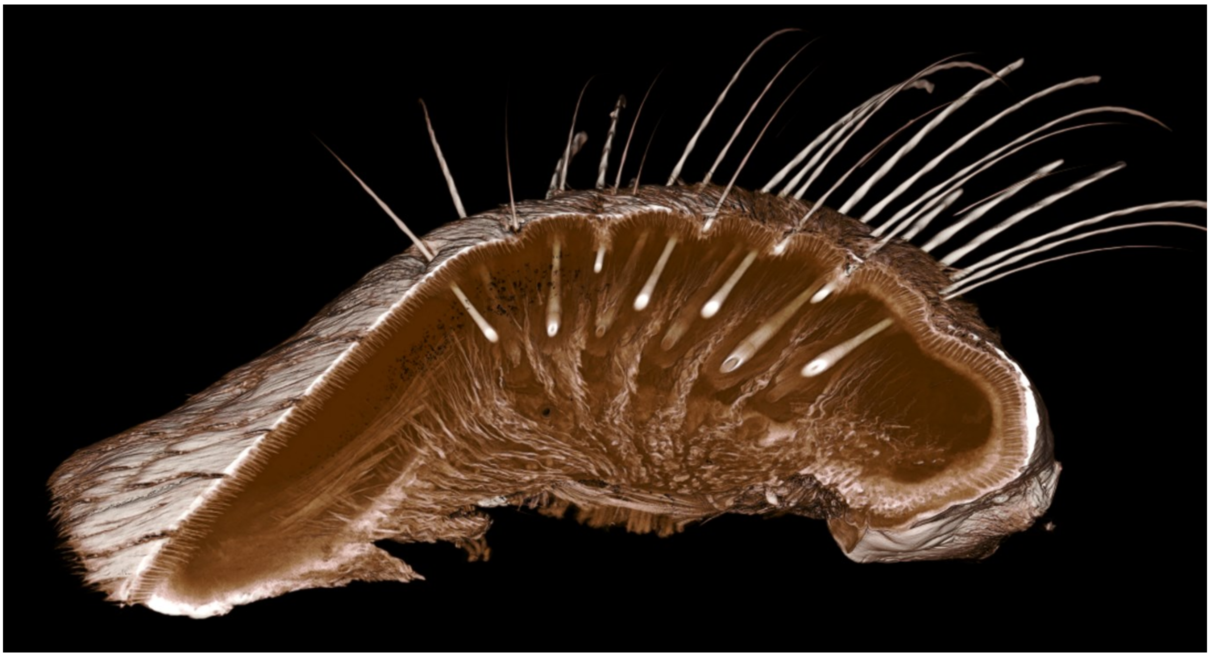

Figure S1: **Orthoslice of a harbour seal mystacial pad, using diceCT (frontal plane):** Showing the musculature within the pad including the large deep extrinsic muscles and undulating vibrissae.

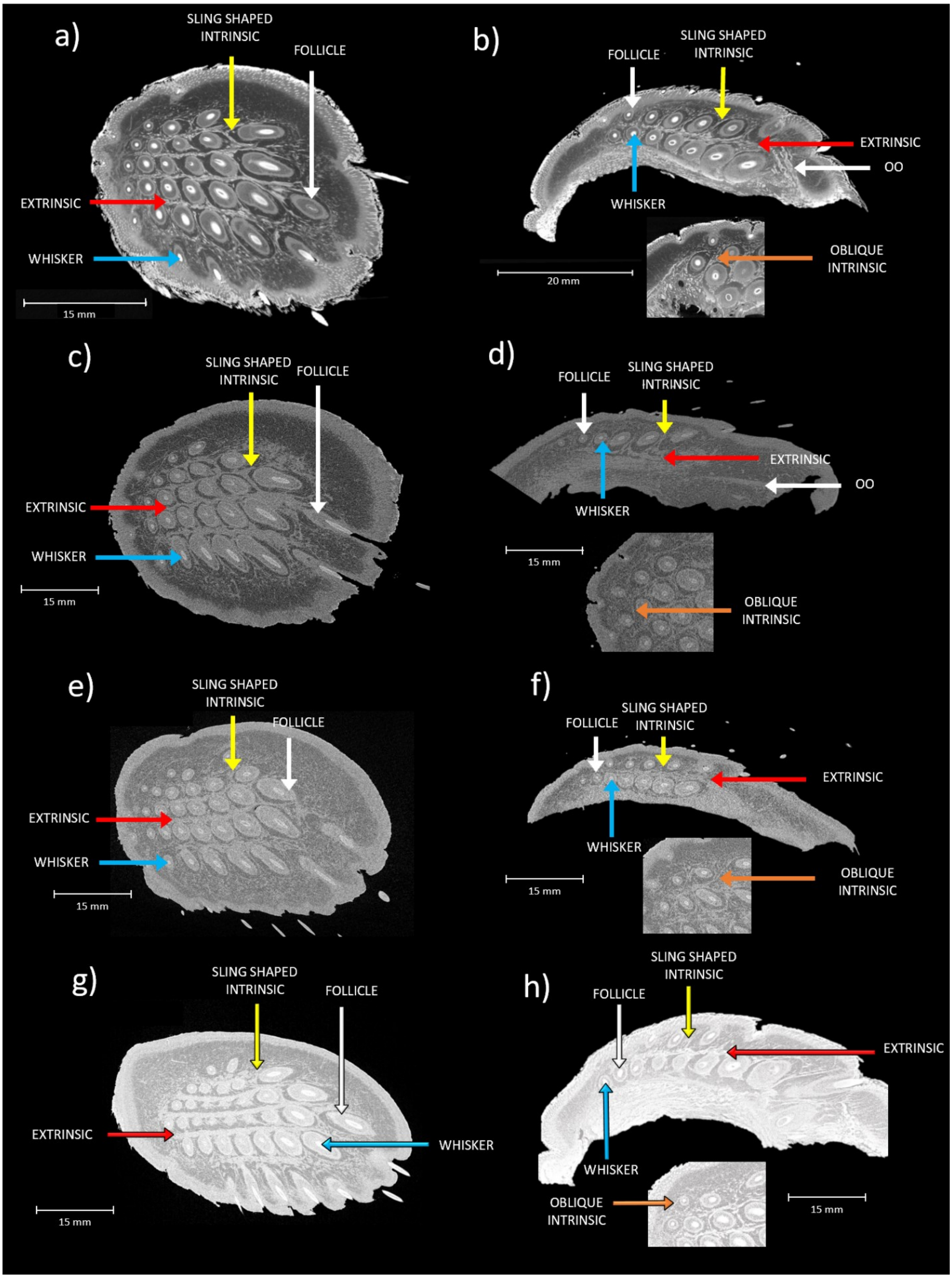

Figure S2: Orthoslices of each of the four harbour seal mystacial pads showcasing musculature: Orthoslice in the sagittal plane (x-y view from vibrissae tips to interior of mystacial pad cheek) showcasing a) and b) harbour seal mystacial pad 04, c) and d) harbour seal mystacial pad 03, e) and f) harbour seal mystacial pad 02 and g) and h) harbour seal mystacial pad 01. Coloured arrows indicate sling shaped intrinsic muscles (yellow arrows), m. maxillolabialis (ML) and m. nasolabialis (NL) extrinsic muscles (red arrows), vibrissae (blue arrows), vibrissal follicles (vertical white arrows), oblique muscles (orange arrows) and OO (horizontal white arrows).

***Table S1:* Vibrissae data for each harbour seal mystacial pad**

Harbour Seal Mystacial Pad	01		02		03		04	
Row	No. of Vibrissae		No. of Vibrissae		No. of Vibrissae		No. of Vibrissae	
	Left Side	Right Side	Left Side	Right Side	Left Side	Right Side	Left Side	Right Side
A	1	0	1	1	2	2	1	-
B	2	2	3	3	4	5	4	-
C	5	5	6	6	6	6	6	-
D	9	8	8	8	8	7	9	-
E	9	9	9	9	9	9	9	-
F	9	9	9	9	9	9	9	-
G	8	8	9	9	9	9	7	-
TOTAL	42		45		47		45	
MEAN	45							

Table S2: Measurements of intrinsic muscle widths across whiskered species: This table presents width measurements of intrinsic muscles from whiskered species. The slice images illustrate intrinsic muscle sections (black arrows) with specific width measurements indicated by a white line and a red arrow. "Species" column identifies the animals by common and scientific names. "Slices" displays portions of images used for muscle measurement. "Width" represents the mean intrinsic muscle width with the standard deviation (SD). "BL" provides the mean body length of the species, and "W/BL" is the mean width of all intrinsic muscles divided by the mean body length for that species. "References" lists the sources for the slice images and body length data.

**Species**	**Slices (µm)**	**Width (mm)**		**Body Length (mm)**	**W/BL (mm)**	**References**
		**Mean**	**±SD**	**Mean**	**Mean**	
Rat (*Rattus norvegicus*)	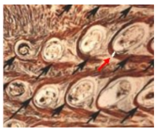	0.1295	0.0870	218.45	0.0006	**Image Slice**: Figure 10a from Haidarliu et al. (2010); Figure 3b from Grant et al. (2016) **Body Length:** Aguh et al. (2013); Porter et al. (2015); Islam et al. (2021)
Mouse (Mus musculus)	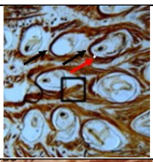	0.1415	0.2395	83.03	0.0017	**Image Slice**: Figure 3a from Grant et al. (2014) **Body Length:** Islam et al. (2021) Slábová and Frynta (2007)
Guinea Pig (*Cavia porcellus*)	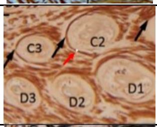	0.0622	0.0814	254.18	0.0002	**Image Slice**: Figure 3a from Grant et al. (2016) **Body Length:** Egena et al. (2010); Barba et al. (2018); FaÃ¯hun et al. (2019)
Opossum (*Monodelphis domestica*)	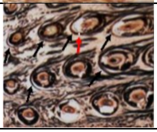	0.1323	0.1857	157.67	0.0008	**Image Slice**: Figure 1e from Grant et al. (2013) **Body Length:** Fadem & Rayve (1985). Redford & Eisenberg (1992); Macrini, (2004)
Harbour Seal (*Phoca vitulina*)	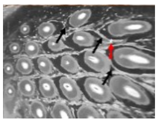	0.3912	0.1644	1612.5	0.0002	**Image Slice**: From Supplementary Figure 2a, c, e and g **Body Length:** McLaren (1993); Lowry et al., (2001); Hutchinson et al. (2016); Harding et al. (2018)

Footnote: To assess intrinsic muscle widths, slices were selected from various journal sources, as listed in Table S2. Measurements were obtained from the rat (*Rattus norvegicus*), guinea pig (*Cavia porcellus*), opossum (*Monodelphis domestica*), house mouse (*Mus musculus*), and harbour seal (*Phoca vitulina*). Using ImageJ software (version 2.14.0/1.54f, Java 1.8.0_322, 64-bit), measurements were calibrated to the scale bars present in each slice. Each visible intrinsic muscle was measured twice, and the mean width calculated. Rows or columns where two intrinsic muscles could not be measured were excluded. To account for body size, muscle widths were normalised by dividing by the mean body length of each species, based on studies listed in Table S2. Our results show that widths of the intrinsic muscles in harbour seals are similar to other species. However, because of the size of a harbour seal we expected larger intrinsic muscle widths, and due to the greater resistance encountered in water, requiring larger muscles for vibrissae protraction.

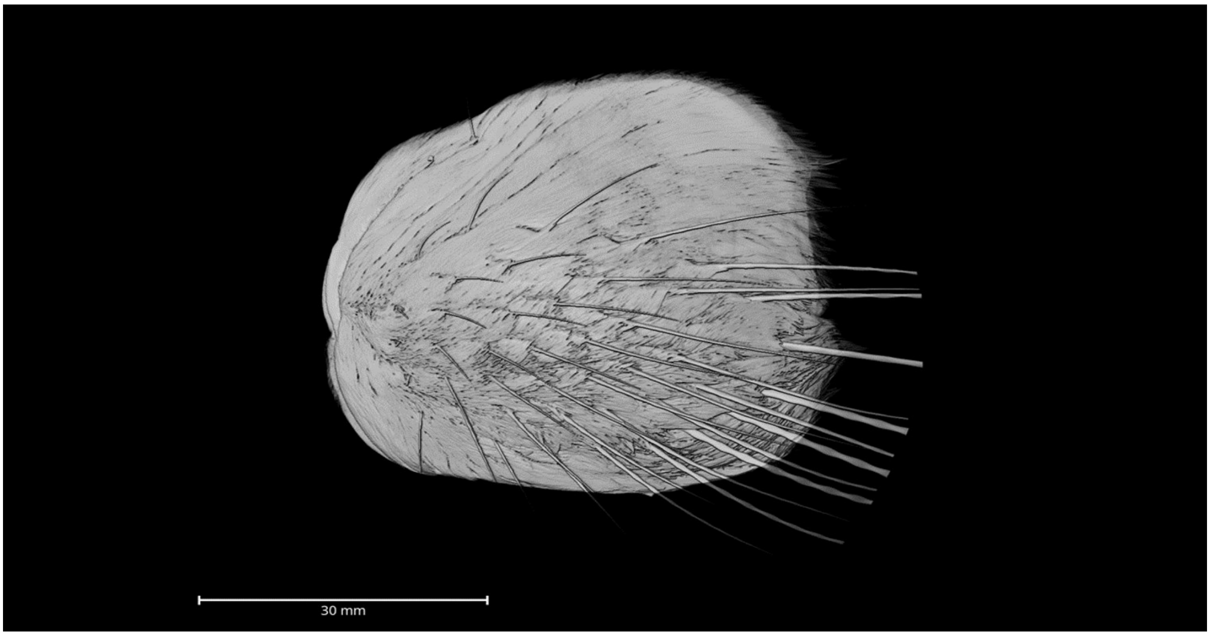

Movie S1: **3D rotation of harbour seal mystacial pad**: A translucent rendering of the mystacial pad which fades out to focus on a single vibrissal follicle. The follicle and the surrounding muscle tissue are coloured as for Figure 5b. The animation shows how the sling shaped muscles wrap around the underside of the follicle and the geometry of the m. maxillolabialis which separate the rows of vibrissae.

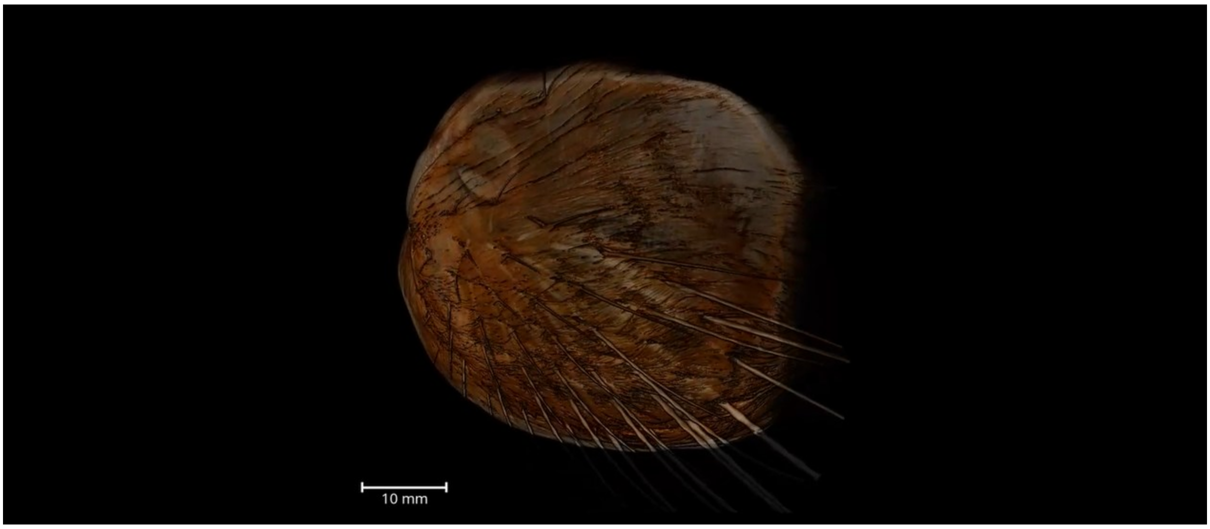

Movie S2: **3D rotation of slicing through the orthogonal planes within harbour seal mystacial pad:** A false colour translucent rendering of the mystacial pad which is clipped in the three orthogonal planes to reveal the internal structure and positioning of vibrissae follicles. The video highlights the curved nature of the pad as no one slice through a plane gives a true cross-section of the muscle structure around the follicles. Also provides the reader unannotated data without authorial interpretation. Structures visible include the vibrissae, follicles, intrinsic muscles and extrinsic muscles.

References

1. Aguh, BI, Yahayab, A,Saidu, IA, Ayeku, PO, and Agba AA (2013). Correlation of body weight and other morphometric measurements in Albino rats (*Rattus Norvegicus*). Scientific Journal of Biological Sciences, 2(3), 39-44. https://www.europub.co.uk/articles/-A-1079
2. Barba, L., Sánchez-Macías, D., Barba, I., & Rodríguez, N. (2018). The potential of non-invasive pre- and post-mortem carcass measurements to predict the contribution of carcass components to slaughter yield of guinea pigs. Meat science, 140, 59–65. https://doi.org/10.1016/j.meatsci.2018.02.019
3. Egena, SS, Husseini, G, Silas, T & Musa, TC (2010). Effect of sex on linear body measurements of guinea pig (*Cavia porcellus*). AU Journal of Technology, 14, 61-65.
4. FaÃ¯hun, AML, Zoffoun, AG, Dedehou, VFGN & Hounzangbe-Adote, MS (2019). Determination of body weight from morphometric characteristics of guinea pigs (*Cavia porcellus*) reared in southern Benin. International Journal of Livestock Production, 10(1), 9-13.

5. Fadem and Rayve (1985). Characteristics of the oestrous cycle and influence of social factors in gray short-tailed opossums (*Monodelphis domestica*). Journal of Reproduction and Fertility 73:337-342.
6. Grant RA, Haidarliu S, Kennerley NJ and Prescott TJ (2013). The evolution of active vibrissal sensing in mammals: evidence from vibrissal musculature and function in the marsupial opossum. J Exp Biol. Vol: 216 (18) p. 3483-94.
7. Grant, RA, Sharp, PS, Kennerley, AJ, Berwick, J, Grierson, A, Ramesh, T & Prescott, TJ (2014). Abnormalities in whisking behaviour are associated with lesions in brain stem nuclei in a mouse model of amyotrophic lateral sclerosis. Behavioural brain research, 259, 274–283. https://doi.org/10.1016/j.bbr.2013.11.002
8. Grant RA, Delaunay MG and Haidarliu S (2016). Mystacial whisker layout and musculature in the Guinea pig (*Cavia porcellus*): A social, diurnal mammal. The Anatomical Record.
9. Haidarliu S, Simony E, Golomb D and Ahissar E (2010), Muscle Architecture in the Mystacial Pad of the Rat. Anat Rec 293: 1192-1206. https://doi.org/10.1002/ar.21156
10. Harding, KC, Salmon, M, Teilmann, J, Dietz, R & Harkonen, T (2018). Population Wide Decline in Somatic Growth in Harbor Seals-Early Signs of Density Dependence. Frontiers in Ecology and Evolution, 6:59.
11. Hutchinson, E, Atkinson S and Hoover-Miller A (2016). Growth and reproductive tracts from fetal to adult harbor seals in the Gulf of Alaska. Marine Ecology Progress Series 557:277-288. DOI: 10.3354/meps11832
12. Islam, MM, Farag, E, Mahmoudi, A, Hassan, MM, Atta, M, Mostafavi, E, Alnager, IA, Farrag, HA, Eljack, GEA, Bansal, D, Haroun, M, Abdeen, R, Al-Romaihi, H, Al-Zeyara, AA, Almalki, SA, & Mkhize-Kwitshana, Z (2021). Morphometric Study of Mus musculus, *Rattus norvegicus*, and *Rattus rattus* in Qatar. Animals : an open access journal from MDPI, 11(8), 2162. https://doi.org/10.3390/ani11082162
13. Lowry, LF, Frost, JK, Ver Hoef JM and DeLong. RA (2001). Movements of satellite-tagged subadult and adult harbor seals in Prince William Sound, Alaska. Marine Mammal Science 14(4):835-861.
14. Macrini, (2004). Monodelphis domestica. Mamm. Species 760, 1-8.
15. McLaren (1993). Growth in pinnipeds. Biological reviews of the Cambridge Philosophical Society, 68(1), 1–79. https://doi.org/10.1111/j.1469-185x.1993.tb00731.x
16. Fleur H. Porter, Federico Costa, Gorete Rodrigues, Helena Farias, Marcelo Cunha, Gregory E. Glass, Mitermayer G. Reis, Albert I. Ko, James E. Childs, Morphometric and demographic differences between tropical and temperate Norway rats (*Rattus norvegicus*), Journal of Mammalogy, Volume 96, Issue 2, 25 April 2015, Pages 317–323, https://doi.org/10.1093/jmammal/gyv033
17. Redford and Eisenberg (1992). Mammals of the Neotropics: the southern cone. Chile, Argentina, Uruguay, Paraguay. University of Chicago Press, Illinois 2:1-430.
18. Slábová M and Frynta D (2007). Morphometric variation in nearly unstudied populations of the most studied mammal: the non-commensal house mouse (*Musmusculus domesticus*) in the Near East and Northern Africa Zool. Anz., 246, pp. 91-101, 10.1016/j.jcz.2007.02.003
