# Supplementary figures and images for: Describing the musculature of mystacial pads in harbour seals (Phoca vitulina) using diceCT
Source: J Anat. 2024 Oct 15;246(5):696–708. doi: 10.1111/joa.14158 (PMC11996717; doi:10.1111/joa.14158)

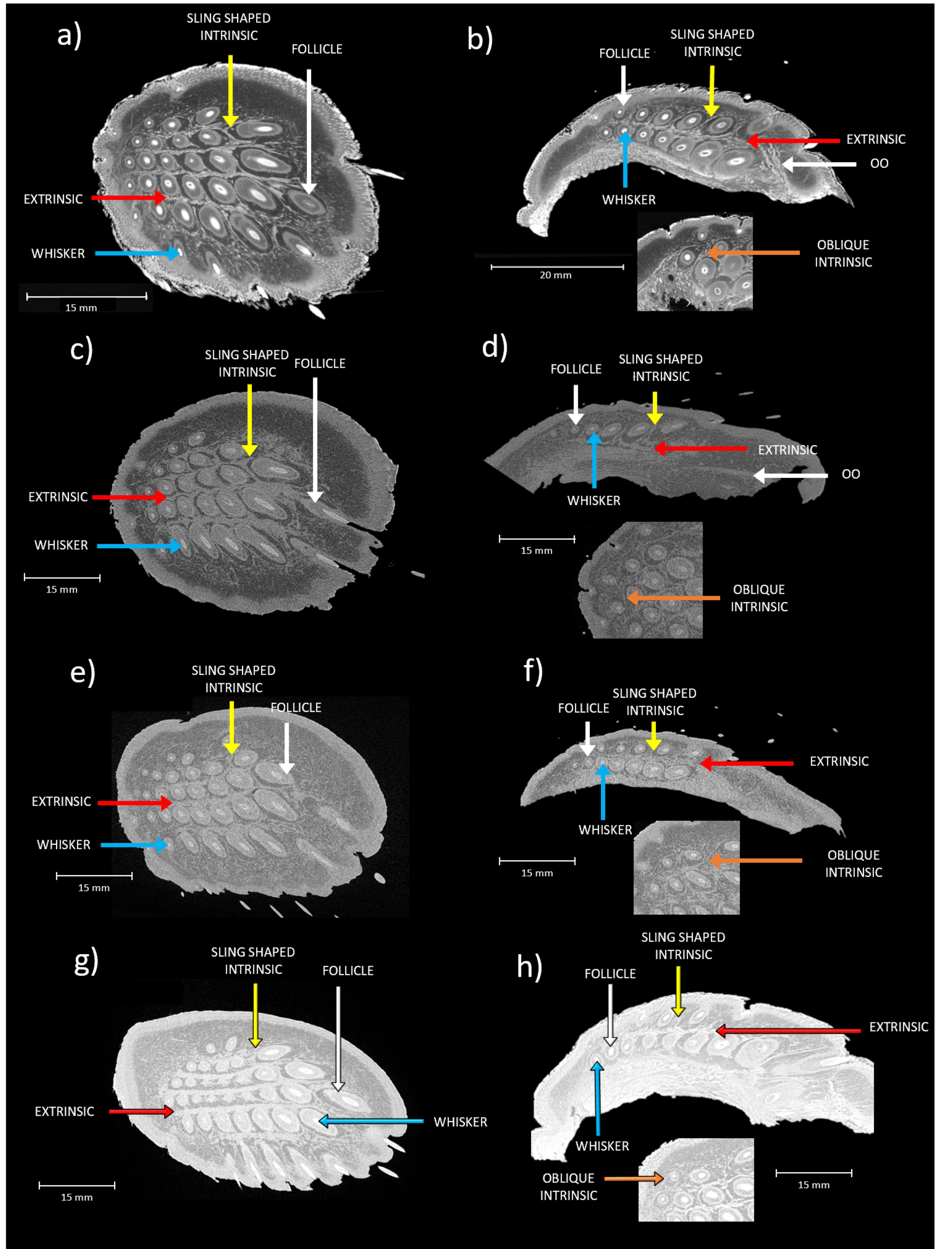

Supplement: Supplementary file 1 — Data S1: [file JOA-246-696-s003.zip › RESUB SUPP MAT FIG 2 MYSTACIAL PADS.tif]

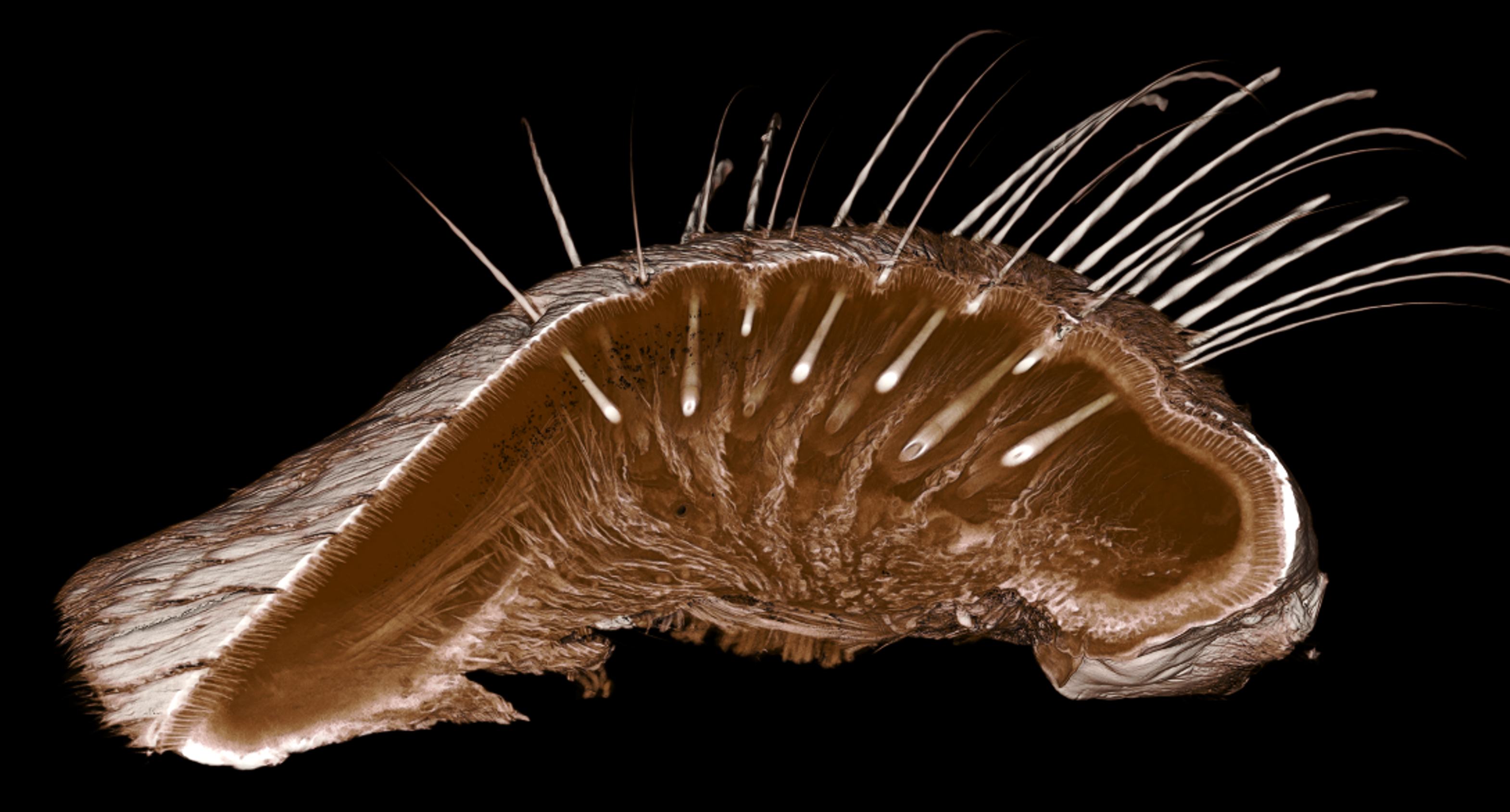

Supplement: Supplementary file 1 — Data S1: [file JOA-246-696-s003.zip › RESUB SUPPLEMENTARY MATERIAL FIG 1 PAD ORTHOSLICE.tif]
